# Feasibility within-subject RCT of neuromuscular electrical stimulation; an Intervention to Maintain and improve neuroMuscular function during period of Immobility (IMMI)

**DOI:** 10.1007/s41999-024-01133-4

**Published:** 2025-01-08

**Authors:** Helal B. Alqurashi, Tahir Masud, Adam Lee Gordon, Mathew Piasecki, Dominic O’Connor, Katie Robinson, John R. F. Gladman

**Affiliations:** 1 School of Medicine, University of Nottingham, Nottingham, UK; 2 Department of Physical Therapy, Faculty of Applied Medical Science, Taif University, Taif, Saudi Arabia; 3 NIHR Nottingham Biomedical Research Centre (BRC), Nottingham, UK; 4 Nottingham University Hospitals NHS Trust, Nottingham, UK; 5 Wolfson Institute of Population Health, Queen Mary University of London, London, UK; 6 Academic Centre for Healthy Ageing, Barts Health NHS Trust, London, UK; 7 School of Health Sciences, University of Nottingham, Nottingham, UK; 8 NIHR Applied Research Collaboration (ARC) East Midlands, Leicester, UK

**Keywords:** Fragility fracture, Sarcopenia, Neuromuscular electrical stimulation, Physical function, Feasibility trial

## Abstract

**Introduction:**

Neuromuscular electrical stimulation (NMES) is a potentially effective intervention to improve outcomes after a fragility fracture, but its feasibility in this group has not been established.

**Methods:**

A feasibility study was conducted in two phases: 1) in the hospital only, and 2) hospital, rehabilitation centres, and participants’ homes. Patients with fragility fracture were randomised to receive NMES for 6 weeks/discharge either to the right or left leg, with the other leg serving as control. Patients who had no mobility issues had contraindications to NMES and were unable to give consent were excluded. NMES was applied to quadriceps and tibialis anterior muscles for 30 min, 3 days/week. Sessions were progressed to achieve 60 min, 5–7 days/week. Feasibility outcomes included participant characteristics, recruitment rate, tolerability, and number of NMES sessions. Clinical outcomes included muscle strength, and ADL at six months.

**Results:**

Overall, 1052 patients were identified, of whom 113 (11%) were eligible, and 29 (3%) were recruited (median Clinical Frailty Score 3, median Barthel ADL score 93/100). The recruitment rate was 0.45/week in phase 1 and 0.9/week in phase 2. Fifty-three percent achieved the target of 24 NMES sessions. However, 5/29 withdrew due to intolerance of NMES. Leg muscle strength improved in both treated and untreated legs, with marginally greater improvement observed in the tibialis anterior of treated legs.

**Conclusion:**

Although it would be feasible to evaluate the specific effect of NMES in fragility fracture patients in a multi-centre trial using home-based NMES, this would be possible only in a minority of mildly frail fragility fracture patients with little premorbid disability.

**Supplementary Information:**

The online version contains supplementary material available at 10.1007/s41999-024-01133-4.

## Key summary points

**Aim:**

Neuromuscular electrical stimulation is a potentially effective intervention to improve outcomes after fragility fracture, but its feasibility in this group has not been established.

**Findings:**

The implementation of neuromuscular electrical stimulation is feasible in a small fraction of fragility fracture patients.

**Message:**

Neuromuscular electrical stimulation should be considered as a supplementary intervention rather than a substitute, with the findings offering insights for future randomised clinical trial design, essential before NMES becomes routine in clinical care.

**Supplementary Information:**

The online version contains supplementary material available at 10.1007/s41999-024-01133-4.

## Introduction

Older people admitted to hospital with fragility fractures frequently develop hospital-acquired disability [1, 2]. This is partly due to loss of muscle mass and function, which develop due to factors including immobilisation, inflammation, and malnutrition [2]. Early rehabilitation using exercise improves outcomes in hospital patients [3–6]. In practice, however, many patients are medically unstable or experience exercise-limiting symptoms that render it unfeasible [7, 8]. An alternative or additional intervention is neuromuscular electrical stimulation (NMES), in which involuntary muscle contraction occurs from non-invasive trains of stimuli to nerves and muscles transmitted through electrodes typically placed over thigh and leg muscles. Patients can use NMES in bed or seated, with or without voluntary effort [9, 10]. A systematic review of 42 randomised clinical trials (RCTs) of NMES in hospitalised adults showed it produced stronger and larger muscles, and better walking, than controls [11]. However, NMES is not yet a routine part of hospital care for patients with fragility fractures. This is partly because applied health research has not yet been conducted to clarify how feasible and acceptable, and clinically effective NMES is in typical clinical practice.

We aimed to evaluate the feasibility of NMES in fragility fracture patients. The objectives of the study were:

1. To determine the proportion of patients with fragility fractures who were willing and able to receive NMES and their characteristics.
2. To determine the acceptability and practicality of NMES in this population.
3. To determine parameters to aid the design of an RCT with clinical outcomes.

## Method

### Methodology

We conducted a cohort study to test recruitment procedures, using a parallel group RCT design for an internal efficacy study in which we randomised participants into two groups: NMES to the left or right leg for 6 weeks with the other leg as control—a within-subject comparison design (split-body RCT). This research was approved by East Midlands — Nottingham 2 Research Ethics Committee (REC reference: 21/EM/0037) in accordance with the ethical standards outlined in the Declaration of Helsinki.

In light of events and our experience, five amendments to our initial protocol [12] were necessary (described and justified in Supplementary data S1). These amendments led to two recruitment phases. Recruitment in the first phase was from patients in the hospital only with the intervention given in the hospital until discharge. Recruitment in the second phase was from hospitalised patients and patients in rehabilitation settings, with the intervention delivered at home for 6 weeks. The amendments also recognised that it would not be possible for us to obtain the required sample size to complete the internal efficacy study (see Supplementary data S1). For the sake of completeness and transparency, the detailed methods and results of the internal efficacy study are given in Supplementary data (S2 and 3).

### Study settings

Participants were recruited in two phases from Nottingham University Hospitals NHS Trust (between October 2021 and mid-February 2022), and then (between June 2022 and the end of November 2022) from that hospital and two care homes with rehabilitation beds run by a rehabilitation provider, Nottingham Citycare.

### Eligibility criteria

#### Inclusion criteria

-  > / = 65 years
- Hospitalised due to incident fragility fracture (hip, spine, pelvis, rib, upper limb, lower limb)

#### Exclusion criteria

- Insufficient mental capacity to give consent
- No new loss of mobility
- Expected to be discharged within 7 days of recruitment (first recruitment phase only)
- Residence too far from the hospital to oversee home NMES sufficiently (second recruitment phase only)
- Unsuitable for rehabilitation because medically unwell, barrier nursed or any other reason
- Unsuitable for NMES (e.g. pacemaker, leg conditions precluding NMES, BMI ≥ 30)
- Unable to communicate sufficiently with a researcher to undertake NMES
- Participant in another research study
- Dysphagia or dialysis (first recruitment phase only)

### Sample size

The sample size in the initial protocol was 60 participants, but this was amended to 30 (see Supplementary data for explanation S1).

### Participant identification and screening

Research staff used a prospectively collected hospital register of all patients with trauma, completed by clinical staff, to identify patients with fractures aged 65 or over with a low-velocity mechanism of injury. Research staff screened potential participants using hospital records, information from ward staff and potential participants.

In the second recruitment phase, rehabilitation staff in the care homes identified potential participants with fractures on a weekly basis. Research staff screened these potential participants and applied the eligibility criteria using care home and hospital records, information from care home staff and potential participants.

### Recruitment

Research staff gave potentially eligible patients a patient information sheet and invited them to participate in the study. Written consent was required.

### Baseline data collection

Baseline data collection comprised:

- Pre-admission Barthel index ADL (BI) and Nottingham Extended ADL (NEADL): assessed for both pre-admission and pre-fracture.
- Clinical Frailty Scale, MUST and Elderly Mobility Scale (EMS) scores: assessed for both post-admission and post-fracture.
- Height and weight
- Handgrip, quadriceps and tibialis anterior (TA) strength
- Ultrasound measures of vastus lateralis (VL) and TA thickness and echogenicity.

See Supplementary data for details S2.

### Randomisation

Participants were randomised, using the “Sealed Envelope” online randomisation service (sealedenvelope.com), to NMES to either the left or the right leg.

### Intervention

After randomisation research physiotherapists, trained in its use, applied NMES to the quadriceps and TA of the randomised leg. Researcher-delivered NMES sessions continued until discharge from the hospital or the rehabilitation facility. In these sessions, research staff applied electrode pads to the skin overlying the muscles. NMES was administered to the assigned leg using NHS approved electrical stimulator device (Premier Combo Plus, Med-Fit Ltd, UK). Four self-adhering surface electrodes were used. Two adhesive electrodes (10*5 cm) were placed on the mid aspect of the quadriceps (5 cm below the inguinal ligament and 5 cm above the patella), and two adhesive electrodes (5*5 cm) were placed on the muscle belly of the TA. An asymmetrical, biphasic, square pulse of 50 Hz, 300 µs pulse duration, for 5 s on and 10 s off was used. The intended session duration was 30 min, 3 times a week, for 6 weeks. The on/off time, and session duration and frequency were progressed weekly to achieve 10 s/10 s, 1 h per day and 5–7 sessions per week, respectively. The target was to achieve a minimum of 24 sessions required [13]. The intensity was adjusted to the maximal intensity tolerated, aiming to achieve visible or palpable contraction.

In the second recruitment phase in which home-based NMES was used, research staff also trained participants in the use of NMES in preparation for continuing at home. The researchers explained how and where to apply the electrodes, and how to operate the NMES device. Discussions included whether the participant had anyone who could help in the process and how they could do so. The researchers provided written information about the use of the NMES device. The researchers gave participants an NMES device to take home and a supply of electrode pads. They observed participants performing self-administered NMES prior to or after discharge, whichever was most convenient. Participants were advised to apply NMES at home to the randomised leg only and to follow the plans for progression. The researchers asked participants to complete a diary recording the date of each session, their duration and maximum intensity, its tolerability and any notes participants wished to make. The researchers provided their telephone numbers in the case of any queries. A researcher contacted participants by telephone (or home visits if required) in the first few days at home and weekly thereafter to reinforce the training and troubleshoot. At the 6-week outcome assessment, research staff offered participants using home-based NMES the opportunity to continue using the NMES device and to apply it to either or both legs.

### Optimisation of home-based NMES and experience

In view of the limited amount of knowledge about the practical use of home-based NMES in older people recovering from fragility fractures, we took an action research approach to optimising the home-based NMES procedures, making observations and enquiries, discussing them with the research team, adjusting the procedures and observing the consequences of doing so.

At the end of the 6-week intervention period, we invited participants to participate in recorded semi-structured interviews to explore their experiences of NMES.

### Outcome assessments

Outcomes of interest comprised:

- Identification, eligibility, consent and recruitment numbers
- Duration of length of stay in hospital
- Number of NMES sessions achieved in each setting (hospital, rehabilitation facility, home), the proportion achieving 24 NMES sessions
- Tolerability of NMES sessions
- Number of participants choosing to continue to use NMES after 6 weeks
- BI and NEADL scores at 6 months
- Internal efficacy study: efficacy outcomes at the end of hospital stay (first recruitment phase) or at 6 weeks (second recruitment phase)—handgrip, quadriceps and TA strength; ultrasound measures of VL and TA thickness and echogenicity.

See Supplementary data for details S2.

### Analysis of action research examination of home-based NMES

In the action research examination of home-based NMES, observations leading to optimisation of the home-based NMES procedures were summarised in narrative form.

Recordings of end of treatment interviews were transcribed verbatim and analysed using both deductive and inductive thematic analysis.

## Results

### Recruitment

**Fig. 1:**
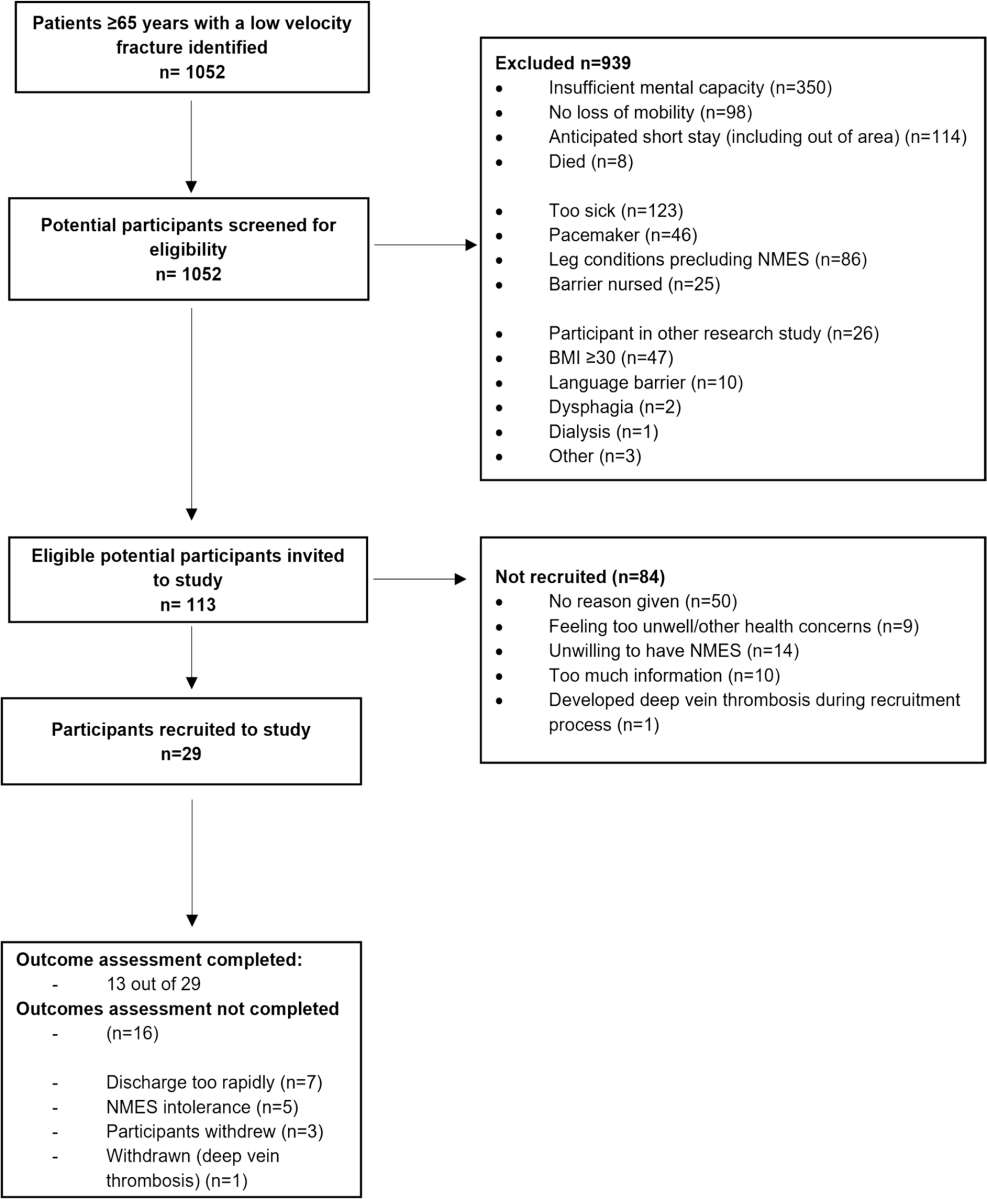
Overall recruitment flow diagram

Twenty-nine participants were recruited. Figure 1 shows the overall recruitment results. Supplementary Figs. 1 and 2 (Supplementary data) summarise recruitment in the two recruitment phases.

Over the 42-week recruitment period, 1052 patients with fragility fractures were identified: 113/1052 (11%) were eligible but only 29 (3%) were recruited. The recruitment rate in the second, 22-week, recruitment phase (20/625, 3%, 0.9 recruits/week) was twice that in the first (20-week) recruitment phase (9/427, 2%, 0.45 recruits/week). Fifty-one patients with fragility fractures were identified from rehabilitation facilities, of whom 13 (25%) were eligible but only 4 (8%) were recruited, partly due to intermittent closure of these facilities due to COVID and infection control procedures. The major reason why recruitment rates were higher in the second recruitment phase was that anticipated short lengths of hospital stay did not preclude enrolment in the study because we provided home-based NMES after discharge.

The main reasons why potential participants were ineligible were cognitive impairment (350/1052, 33%), concurrent medical problems such as being too ill or having contraindications to NMES (330/1052, 31%). We combined leg conditions precluding NMES with contraindications to NMES. Most of these leg conditions were not primarily related to the fractures and included leg hemiparesis or paralysis, skin problems, leg deep vein thrombosis (DVT), leg ulcers, and the use of knee braces. Furthermore, the majority of those eligible (82/113, 73%), did not consent to participate in the study. We did not require potential participants to justify why they declined to give consent, but we recorded what potential participants said when doing so. We judged them to be already overwhelmed by clinical events: only 14/113 (12%) stated that they were unwilling to have NMES.

### Characteristics of participants

The mean age of participants was 79.6 (± 7.1), most of whom had hip fractures, and with mild frailty (median CFS 3), mild mobility limitations at randomisation and little pre-morbid activity limitation. Table 1 summarises the baseline participant characteristics.

**Table 1:** Participant characteristics (*n* = 29 unless stated)

Age mean (sd)	79.6 (7.1)
Sex (male: female)	10:19
Fracture site: hip, other	26, 3
CFS, median (IQR)	3 (2.50–4.00)
Previous Barthel ADL score (mean, SD) (maximum possible 100) (pre-admission)	93.1 (13)
Previous Nottingham ADL score (mean, SD) (maximum possible 22) (pre-admission)	19.55 (3.3)
BMI kg/m2 (mean, SD)	23.4 (3.5)
Handgrip strength, kg, median (IQR)	15.5 (9–21.3)
MUST (post-admission)	Low risk: 21 Medium risk: 5 High risk: 3
Elderly Mobility Scale (0–20) (post-admission), median (IQR)	14 (9–15.5)
Length of stay (hospital recruits, *n* = 25), days, median (IQR)	12 (9–13)

### Intervention delivery

Of the 29 recruited, 16 withdrew during the intervention phase for the following reasons: intolerance of NMES (*n* = 5); development of new contraindication (*n* = 1); other/not stated (*n* = 10).

Eleven of the 20 (55%) recruited in the second phase completed the 6-week intervention protocol. Seventeen of these 20 returned diaries: the median total (in hospital, rehabilitation facility and home) number of NMES sessions was 25 (range 1–78) and 9/17 (53%) achieved 24 or more NMES sessions. The maximum quadriceps and TA muscle stimulation intensities participants achieved (median (range)) were 50 mA (17–99 mA) and 39 mA (18–99 mA) respectively (intensities could range from 0 to 100 mA). The median (range) quadriceps and TA muscle discomfort levels were 2 (0–9) and 3 (0–9) respectively (0 = no discomfort, 10 = maximum discomfort).

Five out of eleven participants chose to continue using the NMES after 6 weeks.

### Follow-up

End-of-intervention efficacy outcomes was assessed on 13/29. In the first phase, 7 participants did not have pre-discharge assessments because they were discharged without warning. In phase 2, 9 participants were not reassessed at 6 weeks after NMES due to withdrawal and loss of follow-up. Furthermore, some data were missing even though the participants completed some of the pre-discharge or post 6-week assessments. The missing data for muscle strength were mainly because of pain related to surgery, whereas the missing data for the ultrasound parameters were due to technical reasons or when the scan area was the site of surgery. The results of the internal efficacy study, which was discontinued due to inadequate recruitment and follow-up, are given in Supplementary data S3.

BI and NEADL scores at 6 months were obtained for 15/29 and 15/29 participants respectively. The mean (SD) (median [IQR]) BI and NEADL scores at six months were 90.67 (13.5) (100 [75–100]) and 18.6 (4.66) (20 [18–21]) respectively.

Only 4 participants were readmitted to the hospital and none died.

### Optimization of home-based NMES

During the action-research process to optimise home-based NMES, we found that our treatment protocol largely worked as intended. The main issue we identified was related to stimulation of tibialis anterior which could be uncomfortable and where it was sometimes difficult to observe a visible contraction and hence determine whether stimulation was being adequately performed. These issues were resolved by:

- Ensuring correct positioning of the electrode pad by doing so lying down
- Reducing the size of the skin–electrode pad
- Relaxation exercises prior to NMES
- Not stimulating VL and TA at the same time

These issues are elaborated in Supplementary data S4.

### Experience of NMES

Eleven participants completed interviews about their experiences: all had completed the full 6-week intervention. Six themes were evident: four that were pre-specified (acceptability, safety, practicality, user experience) and two that emerged inductively (training and support, how and when). The themes are summarized below. Further details and illustrative quotes are given in Supplementary data S5.

#### Acceptability and feasibility

Two participants stated that using NMES initially was awkward. All participants would be willing to use the device if advised to do so clinically (outside a research study). Reasons given for missed sessions were not related to the NMES itself, such as being unwell or on holiday.

#### Safety

No safety concerns for NMES were identified.

#### Practicality

All interviewees quickly overcame any initial unfamiliarity with the application of NMES, although some remained unsure whether they had positioned the electrodes correctly. Some participants had difficulty applying the electrode to TA and required help from another person. Three interviewees suggested that NMES should be delivered alongside an exercise programme: interviewees provided little evidence of any such rehabilitation.

#### User experience

Several interviewees reported that they felt that the intervention had improved their muscle strength, mobility, and pain. Others were unsure, and none felt that NMES made them weaker. Three participants found treatment boring and restrictive.

#### Support and training

Most but not all participants felt that the level of support we provided was adequate.

#### How and when

A wide variety of variations were used (bed/chair, morning/afternoon, 30/60 min) but most used 30-min sessions.

## Discussion

We found that only a minority of fragility fracture patients could be recruited and, in these, only half achieved the target number of NMES treatment sessions. These findings limit the feasibility of a future trial of NMES in this patient group. We initially found that very few hospital patients with fragility fractures were suitable for in-patient NMES. The lengths of hospital stay were so brief that NMES in hospital alone is unlikely to be of value in our hospital: we expect this will be true elsewhere in the UK NHS and other health systems where acute hospitals do not provide rehabilitation. However, we subsequently found that a larger proportion of patients with fragility fractures were suitable for NMES when there was the option of delivering it at home. Nevertheless, a minority (11%) of patients with fragility fractures were eligible and a smaller minority (3%) entered this study – these tended to be pre-frail or mildly frail with little pre-injury disability. The low recruitment rate was primarily due to the fact that most fragility fracture patients did not meet our eligibility criteria, with more than half being excluded because they were too ill or cognitively impaired. Furthermore, only just over half of participants managed to achieve the target of 24 NMES sessions over 6 weeks.

We think it unlikely that there is scope to markedly increase the proportion of fragility fracture patients in whom NMES is clinically applicable and in whom recruitment to an NMES research study is possible. Although there is no absolute reason why patients with cognitive impairment should not use or benefit from NMES, considerable further research and resources would be required to do this safely and ethically. Nevertheless, as 11% of all patients were eligible for this study, this represents a substantial number of fragility fracture patients who could potentially benefit from it.

Our internal efficacy study reported in the Supplementary data was unsuccessful. There were limitations in our use of a within-subject design in which we treated one leg with NMES and left the other untreated as a control. Whilst this design improves the ability to detect treatment effects by reducing between-subject variation, it does not have external validity because in clinical practice an attempt would be made to apply NMES to both legs if possible or to either if one leg has a contraindication such as a DVT or cellulitis. Furthermore, a treatment effect of NMES in one leg could lead indirectly to a treatment effect in the other leg, thereby reducing the treatment effect estimated by comparing the two legs. The high drop-out rate in our study produced a risk of ascertainment bias. The small sample size produced a highly imprecise estimate of the treatment effect. More reliable estimates of effectiveness come from our systematic review of NMES in hospitalised adults, which showed it to be a promising intervention [11], producing a small but statistically significant increase in muscle strength.

Even though the intervention tested in our study was not the same as would be used in clinical practice, the current study has shown that home-based NMES is feasible, albeit only in a minority of pre/mildly frail, minimally disabled fragility fracture patients. Given the review findings and lack of evidence of any major adverse effects, it is reasonable for selected individual patients with fragility fractures to consider using NMES to augment (but not replace) their rehabilitation programme.

Given the resources required to apply NMES routinely in clinical care and the remaining uncertainty that such efforts are justified by substantial health gains, we believe that further research is required before NMES should be a part of routine clinical care. Moderate clinical treatment effects in a future RCT of 7/100 BI and 2/22 NEADL points are plausible by comparison with other rehabilitation trials [14–16] but to detect such effects with *p* = 0.01, and power = 0.90 would require a sample size of approximately 250 [17, 18]. Given that we were able only to recruit 0.9 participants per week for our study, such a study would need to be multi-centred. Despite being expensive and time consuming, such a study would remain of limited value because only a fraction of the minority of eligible fragility fracture patients could be included. Instead, further applied health research could optimise the selection of patients suitable for NMES and optimise treatment programmes for this minority.

Further mechanistic research is also justified to identify and optimise the optimal stimulation parameters to improve muscle strength, and to examine how the intervention interacts with exercise programmes and other interventions to improve outcomes such as vibration therapy, nutritional therapy and anabolic steroids [19–22]. Further technical research is also justified such as to explore further the value of different methods of NMES such as whole-body suits [23].

## Supplementary Information

Below is the link to the electronic supplementary material.

Supplementary file1 (DOCX 251 KB)

## Data Availability

The data supporting the findings of this study are accessible upon request from the corresponding author.
