# Supplementary material for: Feasibility within-subject RCT of neuromuscular electrical stimulation; an Intervention to Maintain and improve neuroMuscular function during period of Immobility (IMMI)
Source: Eur Geriatr Med. 2025 Jan 8;16(2):635–43. doi: 10.1007/s41999-024-01133-4 (PMC12014770; doi:10.1007/s41999-024-01133-4)
Supplement: Supplementary file 1 — Supplementary file1 (DOCX 251 KB) [file 41999_2024_1133_MOESM1_ESM.docx]

Supplementary data

**S1. Amendments to initial protocol**

In our initial protocol we had planned concurrently to evaluate a novel high protein ice-cream supplement in a randomised factorial design. We had also included intramuscular electromyography (iEMG) measurements at baseline and follow-up to examine the effect of NMES on muscular innervation and hence explore mechanistic issues. We had anticipated treating participants using NMES while in hospital until discharge. We had estimated the required sample size based upon an internal efficacy study comparing muscle strength between NMES treated and untreated legs.

On five occasions we sought approval from the research ethics committee for six important changes to the study protocol during its conduct:

- The high protein ice cream supplement was not available for evaluation for logistical reasons, related to its preparation, storage and use in the study hospital. In response, we initially aimed to maintain a randomised factorial design using a high protein conventional oral nutritional supplement instead of the high protein ice-cream (amendment 1). However, during the period when recruitment to the study was live, the study hospital introduced a policy to offer nutritional supplements to all patients with fragility fractures, meaning that randomisation to the nutrition supplement was not permissible. In response we amended the protocol again to discontinue the nutritional arm of the study (part of amendment 2).
- During the first month of recruitment to the study we found that recruitment rates were below our target and we sought ways to overcome this. Our research staff felt that some potential participants were discouraged from giving consent to the study by the mention of the insertion of a needle during iEMG measurements. Given that recruitment to the study was already proving difficult and that iEMG measurement would not be part of a future RCT or clinical practice, we amended the protocol to discontinue this element of the study (included in amendment 2).
- Despite amendment 2, we still had inadequate study recruitment after three months. By this time it had become clear that the lengths of stay for those we had recruited were shorter than anticipated (median length of stay post-randomisation 6 days, range 2-13). This made it impossible to achieve the target of 24 sessions: the median number of NMES sessions delivered to the nine participants recruited in the first phases was only 2 (range 1-3). We also found that participants were discharged from hospital rapidly and without warning, and therfore we were often unable to assess their outcomes before discharge: pre-discharge outcome assessments were made in only two of the initial nine participants. Recognising that the delivery and evaluation of NMES as we had initially planned (in hospital only) was unfeasible, we paused recruitment and made two further amendments to the protocol. In amendment 3, we gained permission to recruit participants from rehabilitation settings, where lengths of stay were longer. In amendment 4, we gained permission to permit the use of home-based NMES with the aim of achieving a total treatment duration of six weeks, whether the participant was recruited from hospital or a rehabilitation setting.
- Whilst two months after the application of amendment 4 (home-based NMES) our recruitment rate appeared to have increased, by this time we estimated that we did not have the resources to achieve our initial sample size (60). We decided that we could answer our feasibility questions with a smaller sample size of 30 participants and sought approval to amend the sample size accordingly in amendment 5. By this time we had completed a systematic review and meta-analysis [11] showing evidence of benefit on efficacy outcomes such as muscle strength, meaning that the internal efficacy aspect of our study was of lesser importance than the feasibility aspects.

The need for these amendments has implications for the feasibility of a future study with clinical outcomes of NMES in fragility fracture patients: the study protocol should not include unnecessary mechanistic aspects that threaten to overburden potential participants; in the absence of settings where hospital or rehabilitation facility lengths of stay are in the order of 4-6 weeks, studies should be planned in which NMES is primarily delivered at home.

The table summarises the amendments.

**Supplementary table 1. Amendments**

| **Amendment number** | **Amendment** | **Reason** |
| --- | --- | --- |
| 1 | Conventional high protein oral nutritional supplement to replace novel high protein ice-cream | Novel high protein ice cream unavailable for evaluation |
| 2 | Removal of nutrition arm of the study | Change in hospital policy made randomisation inappropriate |
| 2 | Removal of iEMG measurements in study | In attempt to increase study recruitment |
| 3 | Recruitment from rehabilitation settings | Recruitment in hospital challenging |
| 4 | Use of home based NMES as well as in hospital or rehabilitation settings | To improve feasibility of NMES |
| 5 | Reduction in sample size | Lack of resources to complete study as intended, and lack of need to complete study as intended. |

**S2. Baseline and outcome data collection methods**

Pre-admission Barthel Index ADL (BI):

BI is an assessment tool to evaluate the individual’s basic ADL such as feeding, bathing, grooming, transfer and mobility. The scoring ranges 0 to 100, with higher score indicating greater independence [24].

Nottingham Extended ADL (NEADL):

NEADL is an extended scale to assess the ability of an individual to perform a broader range of activities beyond the basic ADL, such as shopping, cooking, using transportation and more [25]. It is scored on a scale ranging between 0-22, with higher score indicating better independence.

Clinical Frailty Scale

Clinical Frailty Scale assesses and measures the level of frailty in an individual [26]. It ranges from 0 to 9, with each level corresponding to a different degree of frailty from very fit to terminally ill. The score was recorded from the participant’s medical notes.

Malnutrition Universal Screening Tool (MUST)

It is a universal tool to assess and identify individuals who are at risk of malnutrition based on body mass index, unintentional weight loss, and the impact of acute disease on nutritional intake. Based on total score, an individual can be categorised into one of three level: 0=low risk, 1= medium risk, 2 or more= high risk [27]. The score was recorded from the participant’s medical notes.

Elderly Mobility Scale

Mobility was evaluated using the Elderly Mobility Scale, a 20-point assessment tool widely used in the clinical practice of older individuals [28]. It includes tasks such as transfer, balance, support, mobility and functional reach.

Height and weight

Height and weight were recorded from the participant’s medical notes.

Handgrip

Handgrip was assessed by using the Jamar dynamometer, previously validated [29]. The test was performed by having participants hold the dynamometer and encouraging them to squeeze it as tightly as possible, while the base of the dynamometer was supported. It consisted of three attempts, with each attempt lasting approximately 5 seconds of contraction, followed by a 60-second rest period between each attempt. Furthermore, assessments were performed on both the right and left hands.

Quadriceps and tibialis anterior (TA) strength

Quadriceps and TA muscle strength was assessed before and after the intervention by using the hand-held dynamometer (HDD) Layfette manual muscle tester.

Quadriceps strength was evaluated by asking participants to lie supine with a bolster positioned under the knees to allow for a 35-degree of knee flexion, and the HDD was placed perpendicular to the leg, just above the malleoli.

TA muscle strength was tested by instructing participants to lie supine with their ankles in a neutral position, and the HDD was placed on the upper metatarsal region.

During both of these tests, The HDD was held, and an equal force was exerted while the test was performed by the participant. Each of these tests consisted of three attempts, with each attempt lasting approximately 5 seconds of contraction, followed by a 60-second rest period between each attempt. Furthermore, assessments were performed on both the right and left sides of the body for each evaluation.

Ultrasound measures of vastus lateralis and TA thickness and echogenicity

Muscle thickness and echogenicity of both vastus lateralis and TA muscles were assessed using B-mode ultrasonography equipped with an LA523 transducer (MyLab25 Gold; Esaote, Genova, Italy). For the assessment of VL, the images were captured at 50% of the VL muscle length, with the knee joint nearly fully extended [30]. To assess TA, the scans were taken at 20% of the TA muscle length from the mid-sagittal region, while maintaining the ankle joint in a neutral position [31]. The transducer was positioned longitudinally along the mid-sagittal axis of both the thigh for the VL muscle and the leg for the TA muscle, allowing three scans for each muscle. The acquired images were analysed using ImageJ software (Version 1.53a, National Institute of Health, USA) to measure muscle thickness as the distance between the upper and lower aponeuroses, and muscle echogenicity through grayscale histogram analysis of a defined region of interest. For statistical analysis, the mean of the three measurements was calculated, representing a consolidated value for each muscle parameter.

**Supplementary Figure 1.**

**Recruitment flow diagram (first recruitment period, October 2021 – mid February 2022)**


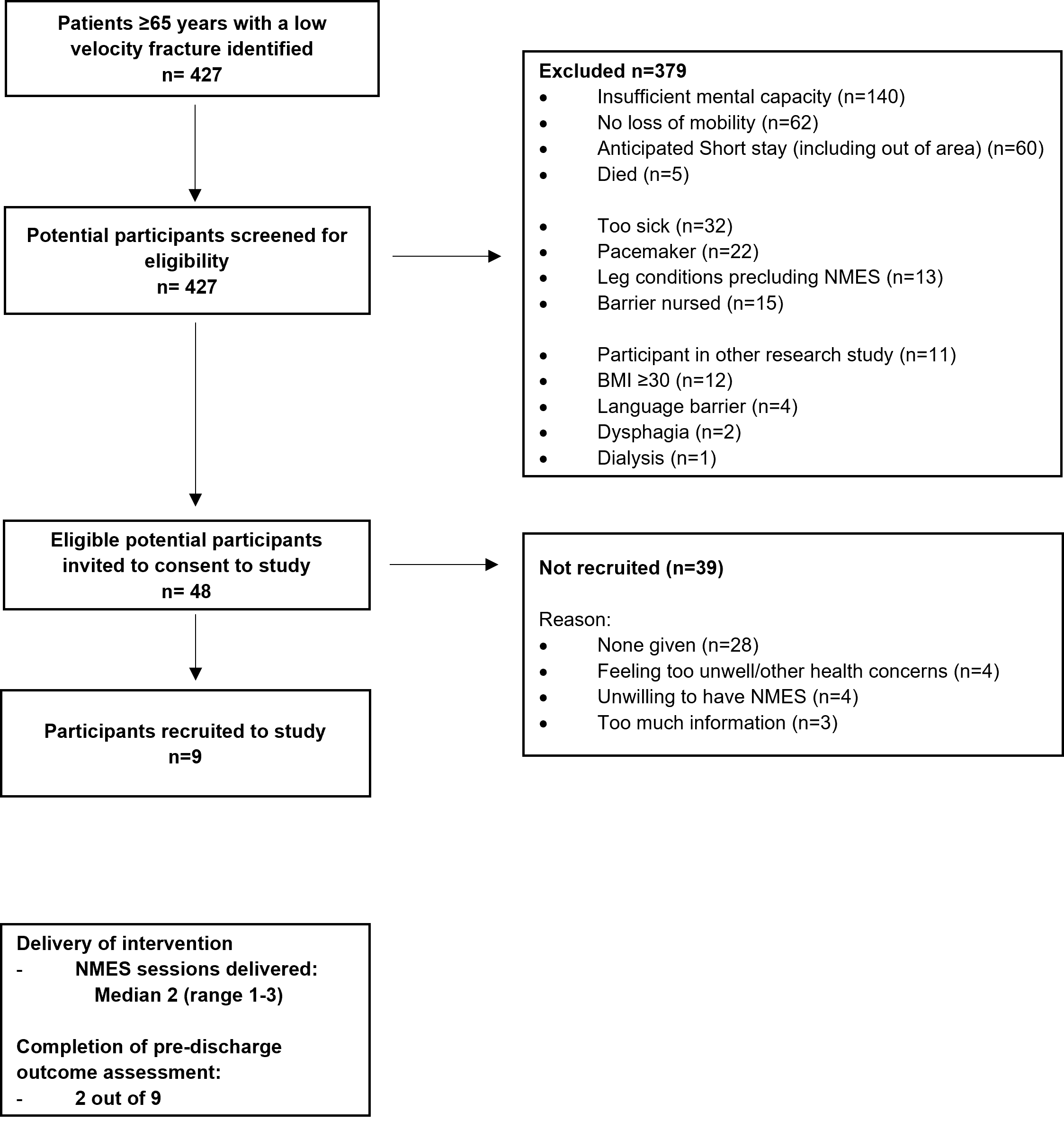


**Supplementary Figure 2.**

**Recruitment flow diagram (Second recruitment period, end June 2022 – November 2022)**


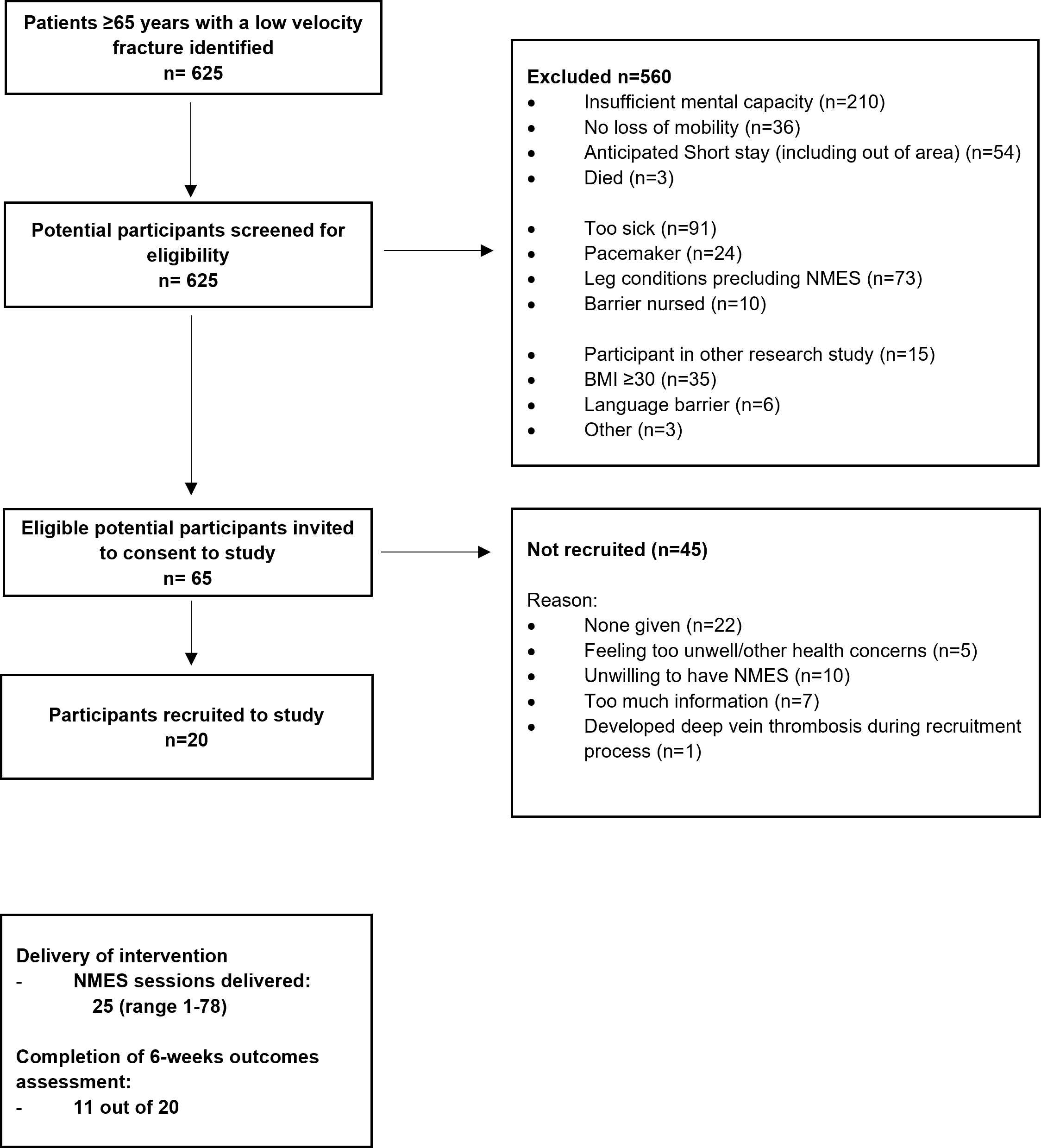


**S3. Internal efficacy study findings**

Method

An internal efficacy study was included within the original study protocol, aiming to compare ultrasound parameters and muscle strengths between randomly allocated NMES-treated and untreated legs. In recruitment phase 1 (when intervention was limited to the in hospital phase), outcomes were ascertained at the end of the hospital stay, but in the second recruitment phase they were ascertained at 6 weeks. Measures were handgrip, quadriceps and TA strength and ultrasound measures of VL and TA thickness and echogenicity.

The statistical analysis for internal efficacy study was to compare changes in muscle parameters between baseline and follow-up in treated and control legs using paired t-tests. Cohen’s effect sizes were calculated (mean change / standard deviation). Effect sizes of <0.2 were considered nil or negligible, 0.2 to 0.49 small, 0.5 to 0.79 moderate, and ≥0.8 large.

Results

As described and justified in “Amendments to initial protocol” we were unable to recruit sufficiently within available resources to complete the internal efficacy study comparing treated and untreated legs. Our findings are given here in for interest and the sake of transparency.

Supplementary Table 2 compares changes in muscle strength for, and between, treated and untreated legs in those for whom paired baseline and outcome data were available.

Supplementary Table 3 compares changes in ultrasound parameters in those for whom paired baseline and outcome data were available.

Supplementary Table 2. Changes in muscle strength

| **Variable** | **Change from baseline to follow-up**  **Control leg** | **Change from baseline to follow-up**  **Intervention leg** | **Control vs Intervention at follow-up** |
| --- | --- | --- | --- |
| **Quadriceps muscle strength**  **Number of cases (n)**  **Mean change (SD), kg**  **Effect size (D)**  **95%CI, kg**  **Significance (paired-t test)** | n= 9  1.1 (1.91)  D=0.58  –0.16 to 1.26  P=0.13 | n= 8  1.5 (2.13)  D=0.7  –0.93 to 1.5  P=0.085 | n= 4  –0.10 (1.22)  D=–0.1  –1.06 to 0.91  P= 0.88 |
| **Tibialis anterior muscle strength**  **Number of cases (n)**  **Mean change (SD), kg**  **Effect size (D)**  **95%CI, kg**  **Significance (paired-t test)** | n= 12  0.7 (1.37)  D=0.5  0.08 to 1.14  P=0.09 | n= 12  0.91 (1.12)  D=0.8  0.14 to 1.45  P=0.017 | n= 11  0.46 (0.55)  D=0.84  0.13 to 1.52  P=0.02 |

**Supplementary Table 3. Changes in ultrasound parameters**

VL: vastus lateralis

TA: tibialis anterior

a.u.: arbitrary units

| **Variable** | **Change between baseline and follow-up Control leg** | **Change between baseline and follow-up**  **Intervention leg** | **Difference in changes between control and intervention legs** |
| --- | --- | --- | --- |
| **VL muscle thickness**  **Number (n)**  **Mean difference (SD), cm**  **Effect size (D)**  **95% CI, cm**  **Significance (paired t-test)** | n= 7  0.13 (0.32)  D=0.4  –0.39 to 1.16  P= 0.33 | n= 6  0.16 (0.41)  D=0.4  –0.46 to 1.21  P= 0.38 | n= 4  0.02 (0.27)  D=0.1  –0.90 to 1.06  P= 0.87 |
| **VL Muscle echogenicity**  **Number (n)**  **Mean difference (SD), a.u.**  **Effect size (D)**  **95% CI, a.u.**  **Significance (paired t-test)** | n= 7  –0.92 (14.11)  D=–0.07  –0.8 to 0.7  P= 0.87 | n= 6  –4.4 (10.85)  D=–0.41  –1.22 to 0.45  P= 0.37 | n= 4  –3.09 (14.09)  D=–0.22  –1.20 to 0.79  P= 0.69 |
| **TA Muscle thickness**  **Number (n)**  **Mean difference (SD), cm**  **Effect size (D)**  **95% CI, cm**  **Significance (paired t-test)** | n= 8  –0.02 (0.13)  D=–0.15  –0.86 to 0.54  P= 0.65 | n= 9  –0.02 (0.16)  D=–0.11  –0.76 to 0.55)  P= 0.75 | n= 8  –0.001 (0.12)  D=–0.008  –0.70 to 0.68  P= 0.98 |
| **TA Muscle echogenicity**  **Number (n)**  **Mean difference (SD), a.u.**  **Effect size (D)**  **95% CI, a.u.**  **Significance (paired t-test)** | n= 8  5.8 (15.04)  D=0.4  –0.35 to 1.1  P= 0.31 | n= 9  2.0 (13.70)  D=0.15  –0.51 to 0.80  P= 0.67 | n= 8  –0.15 (11.20)  D=–0.01  –0.71 to 0.68  P= 0.97 |

Discussion

These results have major limitations. There are limitations in our use of a within subject design in which we treated one leg with NMES and left the other untreated as a control. Whilst this design improves the ability to detect treatment effects by reducing between-subject variation, it has limited external validity because in clinical practice an attempt would be made to apply NMES to both legs if possible or to either if one leg has a contraindication such as a DVT or cellulitis. Furthermore, a treatment effect of NMES in one leg could lead indirectly to treatment effect in the other leg, thereby reducing the treatment effect estimated by comparing the two legs. The high drop-out rate in our study produced a risk of ascertainment bias. The small sample size produced a highly imprecise estimate of the treatment effect.

For these reasons, although our reported treatment effect in tibialis anterior strength (Cohen’s D 0.84) was compatible with a large treatment effect, our results should not be interpreted as providing reliable evidence of such an effect. A more reliable of the possible treatment effect of NMES can be obtained from our meta-analysis of RCTs in similar populations in which we showed, with moderate certainty, that NMES produced a small but significant increase in muscle strength (SMD=0.33, p<0.01) [11].

**S4. Action research findings regarding home-based NMES**

The issues that arose were mainly related to stimulating the TA muscles:

- We initially advised participants to use “synchronous mode” in which both quadriceps and TA muscles were stimulated simultaneously. In this mode, participants reported that the sensation from their quadriceps muscle was so dominant that they could not detect whether TA was optimally stimulated or not. This issue was resolved when participants stimulated the two muscle groups separately.
- Stimulation of TA was initially uncomfortable for most participants. This was resolved by reducing the size of the electrode pad for the TA from 10*5 cm to 5*5 cm.
- Participants found placing the electrodes in position over the TA difficult when in the seating or standing position, due to fear of falling. This was resolved by advising participants either to apply the electrodes while in bed or to ask their family members or carers to help.
- Several participants were unable to feel and see TA muscle contractions and hence determine whether stimulation was adequate. The research team considered that TA contraction is often difficult to detect even in younger people, but the TA tends to be smaller and infiltrated with fat in these people. Trying to resolve that, participants were advised the following:
  - To use relaxation techniques, which might help make contraction more visible, and allow it to contract at lower intensity.
  - To do warm-up exercise before starting NMES session by performing several dorsiflexion movements.

**S5. End of treatment interview findings**

Eleven participants completed interviews of their experiences. They had all completed the full 6-week intervention. Six themes were evident: four that were pre-specified (acceptability, safety, practicality, user experience) and two that emerged inductively (training and support, how and when). Supplementary Table 2 shows illustrative quotes from these interviews.

Acceptability and feasibility

All interviewees found NMES to be acceptable and easy to use and operate, although two participants stated that using NMES initially was awkward. It is worth noting that five participants, who were not interviewed, withdrew because of NMES intolerance. All participants reported that they would be willing to use the device if advised to do so clinically, especially if it would improve their strength. Some participants reported that they missed or stopped sessions because of reasons not related to NMES, such as bad mood, sickness, or social visits and holidays.

Safety

All interviewees reported that there were no harms or side effects after using the NMES.

Some participants reported undesirable feelings during NMES in the ankle and toes during TA stimulation, but these were largely resolved by moving electrode positions. No pain or discomfort was reported at the quadriceps.

Practicality

All interviewees reported that they quickly overcame the initial unfamiliarity with the overall operation and application of NMES (e.g. cable connection, electrode placement, and charging the device).

Electrode placement was the most difficult. Three participants reported some difficulty bending and reaching the TA muscle to attach the electrodes, and required help from a family member. Others were initially unsure whether they found the correct location for electrode placement, and suggested more supervised sessions, and providing pictures and videos might be helpful.

Most interviewees did not require the assistance of a family member or carer, but they were willing to do so if necessary. They felt that older people who live alone and have limited movement might be more likely to require involvement of a family member or carer.

Three interviewees suggested that they should be offered NMES alongside an exercise programme: interviewees provided little evidence of any such rehabilitation.

User experience

Several interviewees reported that they felt that the treatment had improved their muscle strength, mobility, and pain, but others were unsure. None felt that NMES made them weaker.

Three participants found treatment boring, and disliked being unable to walk around during the NMES sessions.

However, some issues were reported but they were related to the hardware. Participants found it difficult to correctly return the electrode pad to its protective packaging. The device we used could stimulate for a maximum of 30 minutes, making sessions of longer duration more complex.

Support and training

Seven participants reported that routine extra researcher visits and training were not required, but should be available on request, but two participants suggested extra visits to troubleshoot and make sure they were applying NMES correctly should be routine. All participants were happy with the level of support we gave - both the educational materials and weekly phone calls. Five participants reported that they did not read or rely on the educational materials, but depended only on the training by the researcher.

How and when

Participants adjusted their treatment within the parameters advised, sometimes in different ways. The majority of participants reported that 30 minutes was enough, and they mostly performed the intervention while they were sitting. However, a few participants found that an hour of NMES can be done, and a few preferred to do it while they were in bed. Additionally, the intervention time during the day varied among participants, some preferring the morning and others the evening.

**Supplementary Table 2. Illustrative quotes**

| Acceptability | *“I’m very, very happy that you saw me in the hospital and you told me about it and I thought it was a very good idea to do this because otherwise I wouldn’t have known about it. And I just hope that it helps other people because there’s such a lot of people having broken bones now isn’t there and it’s not just old people it’s young people as well isn’t it.”* (Female, 78 years)  *“It is a good idea to have one these at home I would say.”* (Female, 78 years)  *“Because I’m hoping it’s going to improve my muscles. I mean I’m very weak on that side and I have been for 30 odd years since I had a laminectomy. And I do, I’ve had a splint for nearly that amount of time because my foot does drop and I’m assuming that when I can’t lift my leg it’s because the muscles are weak in that leg anyway. So if there’s anything that I can possibly use to make it stronger, then yes I’d be up for it.”* (Female, 69 years) |
| --- | --- |
| Safety | *“It doesn’t hurt you or anything like that. It feels quite comfortable and it’s okay when I start to you know move around after I’ve actually done the test, it’s fine.”* (Female, 78 years)  *“I’d say, one day I don’t know, you have good days and you have bad days don’t you. I’m not saying mentally but lack of motivation. Lack of motivation, I thought, oh got to get this out again you know. Is it really worth it? And it’s not saying you’ve got to take your medicine, you’ve got to take your medicine every day whether you like it or not. You see.”* (Male, 88 years)  *“Sometimes I got a sort of tingly feeling, quite a sharp tingly feeling but I found that if I moved the electrode a little, that went away.”* (Female, 89 years) |
| Practicality | *“I found it very easy to use, I didn’t have any problems with it and it’s much better - I get up in the morning and I put it on every day. Accessibility to it is easy.”* (Female, 73 years)  *“I think once you start using it, it’s so self-explanatory, there’s not a lot of parts to it you know. You’ve got the main part which you use to switch it on with and you’ve got the electrodes that go down. I mean, you can’t put them anywhere that they don’t belong. You can’t, you know put them wrong. So yes, that’s fine. Obviously it’s gotten easier the more times I’ve used it.”* (Female, 69 years)  *“If you’ve got a problem with your hip, bending whether you’re lying or sitting to apply the lower leg electrodes is tricky. So it was very difficult.”* (Female, 72 years) |
| User experience | *“Well, I think [laughs] my leg is much stronger and I’m sure the muscle is bigger. I can see that it’s bigger.”* (Female, 89 years)  *“To be honest, I get in and out of bed better, I must admit because I did used to struggle. I just whip my legs over now. It’s hard to get off here because it’s low, but when I get in bed, I’ve noticed I can get in better, so it must have done some good somewhere. I think there is some benefit, yes”* (Female, 85 years)  *“It was definitely- It felt like I was doing something towards helping my recovery, which I appreciated. As well as helping you with your research, so that gave me sort of the inspiration to carry on from using it and doing it.”* (Female, 69 years)  *“Well, I like that you can just sit down when you want to and put the device on and just relax and watch TV while you’re using it.”* (Female, 78 years)  *“It didn’t impact my life, I got into a routine. I just did it as a routine, first thing in the morning generally and then I know it’s done. So I don’t think it impacts your life.”* (Female, 73 years)  *“I think for older people as well, it is beneficial because they can use it at home. They haven’t got to keep going up to the hospital to have this treatment so it is better if they can use it at home.”* (Female, 78 years) |
| Support and training | *“That’s a good thing I think to keep in contact just to say is everything fine. Because if you’ve got a problem, like some people might not have realised what they’ve got to do when its battery was running out. It’s nice you knew you’d be able to ask someone.”* (Female, 73 years)  *“The written instructions, I didn’t really read them. You showed me how to use it”* (Male, 88 years)  *“Well I think it’s just phone call, you mean you don’t have to keep visiting really do you unless there’s a problem but I would think that just the phone call to see how we’re going on and if we have any problems and then we can get back to you.”* (Female, 78 years) |
| How and when | *“Well I could do it for an hour, it’s just setting my goal for the day. So, I’ve been doing it, the majority of the time I’ve been doing every day as opposed to, So 30 minutes for me is a good length of time.”* (Female, 73 years)  *“I think because once you’ve fitted all the electrodes you might just as well do an hour. And most television programmes are an hour anyway [laughs]. So I think an hour is a good time.”* (Female, 89 years)  *“I think 30 minutes because an hour is quite a long time. I mean you might as well have half an hour and you could probably do another half an hour later in the day.”* (Female, 78 years) |

**References**

11. Alqurashi HB, Robinson K, O’Connor D, Piasecki M, Gordon AL, Masud T, et al. The effects of neuromuscular electrical stimulation on hospitalised adults: systematic review and meta-analysis of randomised controlled trials. Age and Ageing. 2023;52(12).

24. Collin C, Wade DT, Davies S, Horne V. The Barthel ADL Index: a reliability study. Int Disabil Stud. 1988;10(2):61-3.

25. Gladman JR, Lincoln NB, Adams SA. Use of the extended ADL scale with stroke patients. Age Ageing. 1993;22(6):419-24.

26. Rockwood K, Song X, MacKnight C, Bergman H, Hogan DB, McDowell I, et al. A global clinical measure of fitness and frailty in elderly people. Cmaj. 2005;173(5):489-95.

27. Elia M RC, Stratton R, Todorovic V, Evans L, K. F. Malnutrition Universal Screening Tool (MUST) for adults. 2004.

28. Smith R. Validation and Reliability of the Elderly Mobility Scale. Physiotherapy. 1994;80(11):744-7.

29. Roberts HC, Denison HJ, Martin HJ, Patel HP, Syddall H, Cooper C, et al. A review of the measurement of grip strength in clinical and epidemiological studies: towards a standardised approach. Age Ageing. 2011;40(4):423-9.

30. Franchi MV, Longo S, Mallinson J, Quinlan JI, Taylor T, Greenhaff PL, et al. Muscle thickness correlates to muscle cross-sectional area in the assessment of strength training-induced hypertrophy. Scand J Med Sci Sports. 2018;28(3):846-53.

31. Martinson H, Stokes MJ. Measurement of anterior tibial muscle size using real-time ultrasound imaging. Eur J Appl Physiol Occup Physiol. 1991;63(3-4):250-4.
